# Children’s Health: Mother’s Thyroid, Baby’s Health

**DOI:** 10.1289/ehp.112-a612

**Published:** 2004-08

**Authors:** M. Nathaniel Mead

Since the 1970s, epidemiologic studies have linked maternal thyroid insufficiency during gestation with fetal brain malformation, fetal death, and miscarriage. The fetus is wholly dependent on the maternal thyroid during the first 10–20 weeks of gestation. U.S. women generally get enough iodine, the elemental nutrient essential for synthesis of the thyroid hormone thyroxine (T_4_). But regular daily intake may not be sufficient during pregnancy due to metabolic changes in the mother-to-be, and recent studies suggest that detection and treatment may be needed long before birth. These and other topics were discussed by scientists at a January 2004 symposium cosponsored by the Centers for Disease Control and Prevention, the National Center on Birth Defects and Developmental Disabilities, and the American Thyroid Association (ATA).

Early maternal thyroidal insufficiency, or EMTI, is a failure of the maternal thyroid to provide an adequate supply of T_4_ in early pregnancy. According to Steven Lamm, a pediatrician and director of the Washington, D.C.–based Consultants in Epidemiology and Occupational Health, EMTI may affect 0.5–5.0% of all pregnant women. When depletion occurs early in pregnancy, fetal brain formation can be markedly altered. Even subtle degrees of thyroid dysfunction in pregnant women might be associated with impaired psychomotor development in their infants, toddlers, and preschool children.

While there’s no doubt that EMTI is related to poor fetal outcomes, the follow-up data on child development are only available until 5–6 years of age, so it’s still unknown whether these developmental delays persist over the long term, said Victor Pop, a professor in the Department of Clinical Health Psychology at Tilburg University, Netherlands, whose landmark study on EMTI was published in the February 1999 issue of *Clinical Endocrinology*. In a later study published in September 2003 in *Clinical Endocrinology*, Pop found that women with the lowest tenth percentile of T_4_ concentrations at 12 weeks’ gestation bore children who experienced impaired mental and motor functioning at age 1–2 years. In EMTI women who showed an increase in T_4_ concentrations at 24 and 32 weeks’ gestation, child development was not adversely affected. Most of the concerns related to fetal risk have focused on the first half of gestation. However, the third trimester is a critical time for cerebellar development and myelination.

The limited amount and quality of the evidence to date is one reason it has been difficult to reach consensus on the etiology as well as screening and treatment requirements for EMTI. Researchers aren’t sure whether using T_4_ to treat women with EMTI benefits all children of these mothers, or whether there are unforeseen effects. Therefore, placebo-controlled studies are urgently needed, said Pop.

John Lazarus, a senior lecturer in medicine at the University of Wales, United Kingdom, described his upcoming randomized clinical study of 22,000 women at 13–16 weeks’ gestation. An experimental group will have T_4_ and the complementary thyroid-stimulating hormone (TSH) measured and thryoxine treatment applied if necessary, while the control mothers will remain untested until after their babies are delivered. Children from both groups will undergo developmental testing at ages 2 and 5 years. This study will rigorously evaluate the impact of both subclinical maternal hypothyroidism and hypothyroxinemia (inadequate TSH and free T_4_, respectively) on the IQ scores of the offspring, as well as the effect of prenatal treatment.

Additional discussions focused on the possible need for screening and treatment. “While it is not yet known whether early identification and treatment of thyroid deficiency will avoid fetal death and neuropsychological deficits in the offspring, it is clear that women themselves will benefit,” said James Haddow, medical director of the Foundation for Blood Research in Scarborough, Maine. “Many women go undiagnosed for longer periods of time, so that they lack the energy they need to function well in everyday life during their child’s early years, when the demands placed on them are greatest.”

Haddow contended that TSH measurement should be added to the list of tests routinely performed at the first prenatal visit (the ATA currently advocates testing for pregnant women with a history of miscarriage, fetal loss, infertility, autoimmune disease, goiter on exam, and family history of thyroid disease). Lamm and other participants also suggested that normal levels for both TSH and T_4_ should be determined for the different stages of pregnancy. Another suggestion was to supplement prenatal vitamins with 150 micrograms of iodine (many currently contain little or none).

But scientists still need to agree on other matters, such as TSH and/or T_4_ cut-off points for defining high risk. A TSH level of 2.5 milliunits per liter was proposed as a good initial cut-off. “This is a conservative cut-off,” said conference co-planner Joseph Hollowell, a professor of pediatrics at the University of Kansas Medical Center, “and it will prompt further investigation to see if there’s a real problem.”

## Figures and Tables

**Figure:**
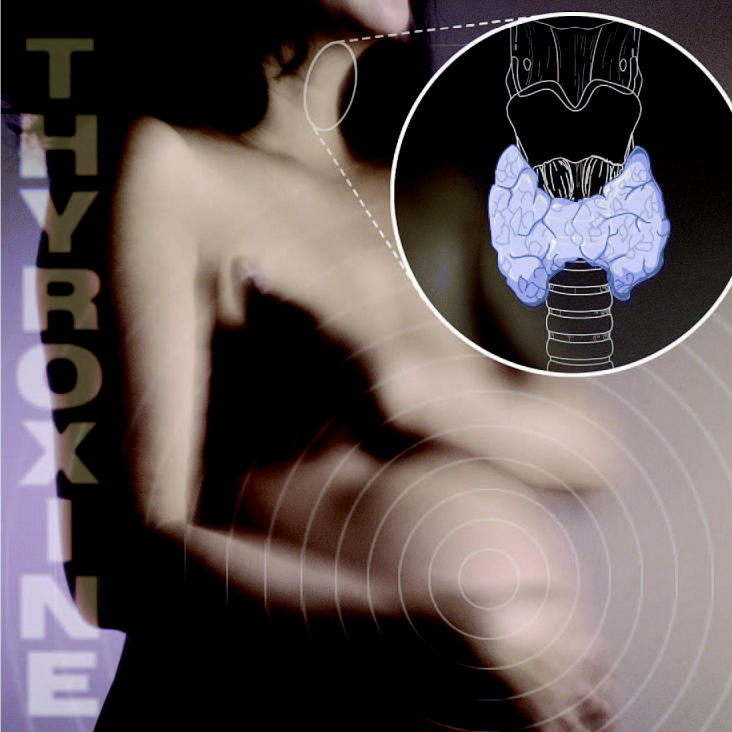
**Intimately connected.** New studies are showing the significance of a healthy thyroid in mothers-to-be on the future health of their babies.

